# Genomic regions associated with stripe rust resistance against the Egyptian race revealed by genome-wide association study

**DOI:** 10.1186/s12870-020-02813-6

**Published:** 2021-01-14

**Authors:** Mohamed A. Abou-Zeid, Amira M. I. Mourad

**Affiliations:** 1 Wheat Disease Research Department, Plant Pathology Research Institute, ARC, Giza, Egypt; 2 Department of Agronomy, Faculty of Agriculture, Assiut University, Assiut, Egypt

**Keywords:** Genome-wide association study, Single marker analysis, Linkage disequilibrium, gene expression, Coefficient of Infection, Disease severity

## Abstract

**Background:**

Wheat stripe rust (caused by *Puccinia striiformis* f. sp. *Tritici*), is a major disease that causes huge yield damage. New pathogen races appeared in the last few years and caused a broke down in the resistant genotypes. In Egypt, some of the resistant genotypes began to be susceptible to stripe rust in recent years. This situation increases the need to produce new genotypes with durable resistance. Besides, looking for a new resistant source from the available wheat genotypes all over the world help in enhancing the breeding programs.

**Results:**

In the recent study, a set of 103-spring wheat genotypes from different fourteen countries were evaluated to their field resistant to stripe rust for two years. These genotypes included 17 Egyptian genotypes from the old and new cultivars. The 103-spring wheat genotypes were reported to be well adapted to the Egyptian environmental conditions. Out of the tested genotypes, eight genotypes from four different countries were found to be resistant in both years. Genotyping was carried out using genotyping-by-sequencing and a set of 26,703 SNPs were used in the genome-wide association study. Five SNP markers, located on chromosomes 2A and 4A, were found to be significantly associated with the resistance in both years. Three gene models associated with disease resistance and underlying these significant SNPs were identified. One immune Iranian genotype, with the highest number of different alleles from the most resistant Egyptian genotypes, was detected.

**Conclusion:**

the high variation among the tested genotypes in their resistance to the Egyptian stripe rust race confirming the possible improvement of stripe rust resistance in the Egyptian wheat genotypes. The identified five SNP markers are stable and could be used in marker-assisted selection after validation in different genetic backgrounds. Crossing between the immune Iranian genotype and the Egyptian genotypes will improve stripe rust resistance in Egypt.

**Supplementary Information:**

The online version contains supplementary material available at 10.1186/s12870-020-02813-6.

## Background

Wheat stripe rust (*Puccinia striiformis* f. sp. *Tritici),* also known as yellow rust*,* is one of the most devastating wheat diseases in the world. It can cause losses in yield from 10 to 100% [1]. New stripe rust races appeared around the year 2000, spread rapidly, and caused significant yield losses [2, 3]. These new races are more aggressive and tolerant to high temperatures than previous races [4]. In Egypt, the appearance of new stripe races resulted in losing the resistance in several of the most resistant cultivars, like Misr_2, Giza_168, and Sakha_61 [5]. Moreover, the lack of genetic diversity among Egyptian wheat cultivars is a serious problem that could increase the virulence of stripe rust causing a huge reduction in wheat production and productivity in Egypt.

Stripe rust could be controlled by the application of fungicides. However, utilization of stripe rust resistant genotypes is a more economically viable and environmentally friendly solution [6]. Furthermore, there is a misuse of the fungicide application by most farmers. Many stripe rust resistance genes were identified with a total number of 74 officially designated and more than 40 temporarily named stripe rust-resistant genes [6–8]. Unfortunately, most of these identified genes are race-specific which makes them ineffective against the new races [9]. For this reason, race non-specific resistance genes are more durable as they are effective against many of the pathogen races. For example, genes *Yr5* [10], *Yr15* [11], and *Yr18* [12] are effective and durable against the new *P. striiformis* races. Therefore, pyramiding durable stripe rust resistance genes are urgently needed and one of the important objectives in breeding programs.

One of the most important steps for the breeding of stripe rust resistance is to identify the resistance genes which are effective under the targeted environment. Identification of resistance genes is an essential step in marker-assisted selection and resistance breeding. Wheat genotyping with the different types of molecular markers such as Diversity Array Technology (DArT) and Simple Sequence Repeated (SSR) helps in identifying the presence of the resistance genes. Furthermore, new sequencing methods such as Genotyping-by-sequencing (GBS) generate several single nucleotide polymorphism (SNP) markers that covering a high percentage of the wheat genome [13–15]. These SNPs provide wheat breeders with more information about the genetic control of stripe rust resistance by using genome-wide association study (GWAS), genomic selection, and genetic diversity studies.

Compared with the traditional QTL mapping and linkage disequilibrium (LD), GWAS produces more resolution association mapping with less time-consuming and more cost-effectiveness [16]. GWAS is a powerful approach that identifies novel alleles associated with complex traits by utilizing linkage disequilibrium and examine marker-trait associations [17–19]. It has been applied successfully to study the genetic architecture of disease resistance in wheat [20–27]. For stripe rust resistance, GWAS was reported as an effective tool to identify a large number of favorable alleles controlling the resistance [28]. Many studies have been done on stripe rust resistance using GWAS and identified many QTLs controlling the resistance distributing across the whole genome [29–31]. In addition, many simple sequence repeats (SSR), sequence-Tagged site (STS), sequence-characterized amplified region (SCAR), diversity arrays technology (DArT), and Kompetitive allele-specific PCR (KASP) markers have been reported as good markers for MAS for stripe rust resistance genes in wheat [32–35]. Furthermore, Maccaferri et al., (2015) reported association mapping of the resistance to the American race of *P. striiformis* using a world-wide collection of spring wheat genotyped by DArT markers. Evaluating wheat genotypes collected from different countries from the world helps in identifying the possible sources of the resistance. As stripe rust resistance started to be broken in Egyptian cultivars, therefore it is urgently needed to look for other sources of resistance in wheat germplasm. However, the evaluated genotypes must be adapted to the Egyptian conditions to be used as parents in breeding programs. In the recent study, we used 103-spring wheat genotypes which were collected from 14 different countries all over the world and are well adapted to the Egyptian environment (Dr. Ahmed Sallam, personal communication). The objectives of this study were to (1) understand the stripe rust resistance in Egyptian wheat and wheat genotypes collected from different countries all over the world, (2) identify allele markers associated with the resistance to stripe rust Egyptian race, and (3) select the best genotypes to be used in future breeding programs to improve wheat resistant to stripe rust.

## Results

**Fig. 1:**
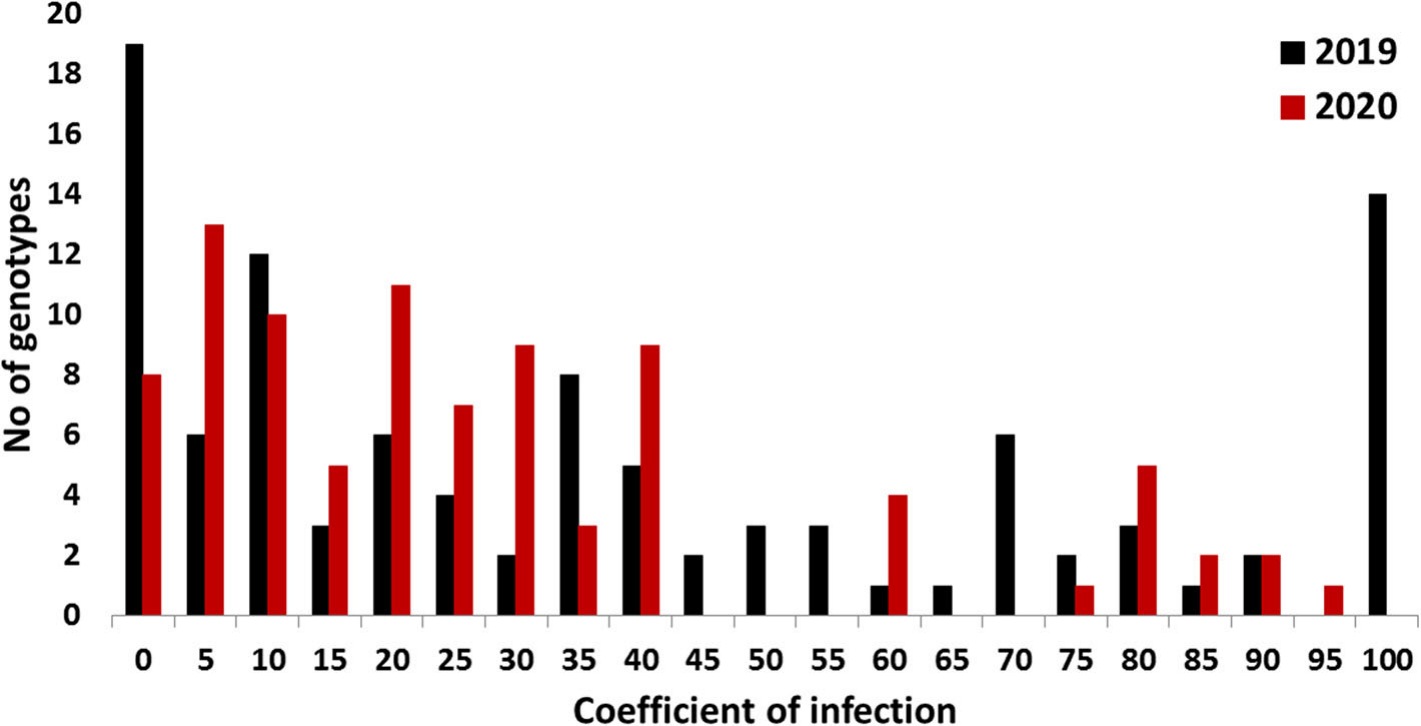
The response of the 103-tested spring wheat genotypes to stripe rust resistance at the adult stage under the Egyptian conditions at 2019 and 2020

To test the analysis of variance of stripe rust resistance, data were transformed using the arcsin root square method. For both years, the Shapiro-Wilk normality test had a highly significant *p*-value of 2.116e^-08^ and 4.129e^-08^ confirming the unnormal distribution of the untransformed data. Compared with the untransformed data, transformed data was better normally distributed (Supplementary figure 1 and 2). The analysis of variance revealed highly significant differences among the genotypes for stripe rust resistance (Table 1). The coefficient of Infection (CI) ranged from zero to 100% and from zero to 95% in 2019 and 2020, respectively (Figure 1). Highly significant differences were found between the years and genotypes x years interaction. Broad-sense heritability was high across the two years (H^2^_B_=0.73).

**Table 1:** Analysis of variance for stripe rust resistance in the 103-spring wheat genotypes

Source	d.f	M.S.
Years (Y)	1	21959**
Replications (R)	2	308.4
Genotypes (G)	102	4675.5**
GY	89	1271.30**
GYR	383	518.26
Heritability	72.8	

*p < 0.05, **p < 0.01

**Fig. 2:**
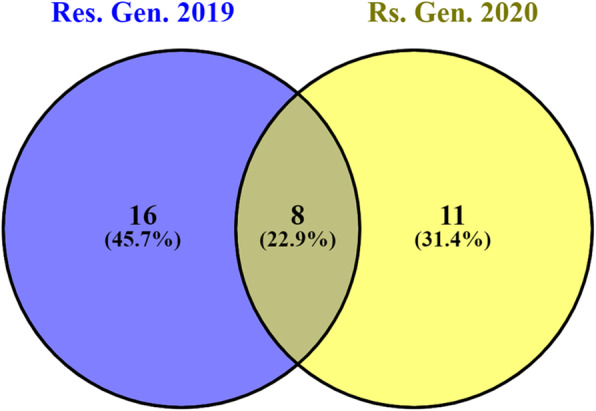
Number of resistant genotypes with coefficient of infection (CI) ranging from 0-4% in 2019 and 2020 experiments

Based on CI, 19 and eight genotypes showed 0% CI in 2019 and 2020, respectively. Also, six and 13 genotypes were resistant with CI four% or less in 2019 and 2020, respectively. The total number of resistant genotypes (with CI ranging from 0-4%) was 24 and 19 genotypes in 2019 and 2020, respectively. Only eight genotypes were resistant to stripe rust in both years (Figure 2 and Table 2). These genotypes are; One Canadian genotype (PI_556465), one Saudi Arabian genotype (PI_574347), two Iranian genotypes (PI_243679 and PI_625253), two Kenyan genotypes (PI_237655 and PI_237658), and two Egyptian genotypes (Misr_1 and Beni Sweif_4).

**Table 2:** The response of the eight resistant genotypes in both years, their original country, and subpopulation based on the result of structure analysis [36]

Res. Gen.	Country.	Sub. Pop.	C.I (2019)	C.I. (2020)
Misr_1	Egypt	Sub. Pop. 1	2.67	4
Beni Swief_4	Egypt	Sub. Pop. 3	0	3.33
PI_556465	Canada	Sub. Pop. 1	0	2.33
PI_625253	Iran	Sub. Pop. 2	4	1.33
PI_243679	Iran	Sub. Pop. 2	0	0
PI_237655	Kenya	Sub. Pop. 1	1.42	2.67
PI_237658	Kenya	Sub. Pop. 1	0	0
PI_574347	Saudi Arabia	Sub. Pop. 1	0	0

### Association mapping for stripe rust resistance

The GBS-SNP markers and population structure

The GBS generated a set of 36,720 SNPs after filtration for MAF >0.05, maximum missing sites per SNP<20%, and maximum missing sites per genotype <20% [20, 21, 37]. Heterozygous loci were marked as missing and the filtration was repeated. As a result of this filtration, a set of 26,703 SNP markers for 102 genotypes was generated. This set was used in the GWAS analysis. The new SNPs distributed across all wheat chromosomes, increasing the possibility of QTL detection.

The structure of the recently studied panel was extensively studied in our previous manuscript [36]. To wrap up, the 103-genotypes were classified into three subpopulations. The eight resistant genotypes were distributed on the three sub-populations indicating that the hybridizations among these genotypes will be very effective (Table 2).

### Genome-wide association study (GWAS) and linkage disequilibrium (LD) between the significant SNPs

**Fig. 3:**
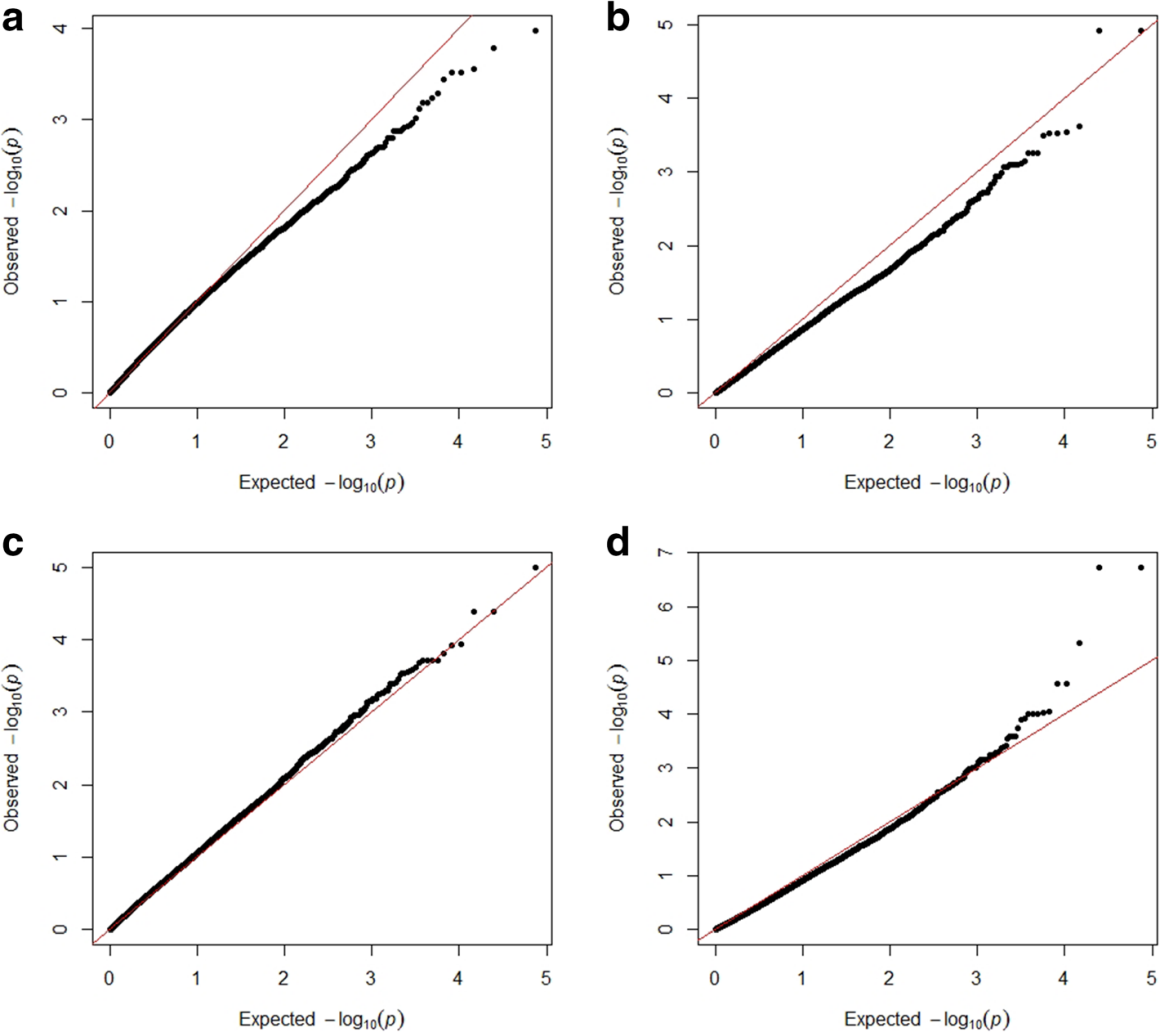
Quantile-Quantile (QQ) plot comparing the performance of the mixed linear model (MLM) used for of GWAS for stripe rust resistance at 2019 **a** and 2020 **b** with the performance of generalized linear model + principle coordinate (GLM +PC) for stripe rust at 2019 **c** and 2020 **d**

Due to the presence of population structure in the studied genotypes, which causes a spurious association, two different models were tested: mixed linear model + kinship (MLM+K) and general linear model + population structure (GLM+PC). The QQ-plot of both models is presented in figure 3. The QQ-plot evaluating the performance of MLM models skewed below the reference line in both years, 2019 and 2020, indicating the overcorrection of the MLM+K model due to the use of the kinship (Figure 3a and b). Instead, the GWAS was performed using GLM+PC in which QQ-plot represented an ideal distribution on the reference line for both the 2019 and 2020 experiments indicating the high correction efficiency (Figure 3 c and d).

**Fig. 4:**
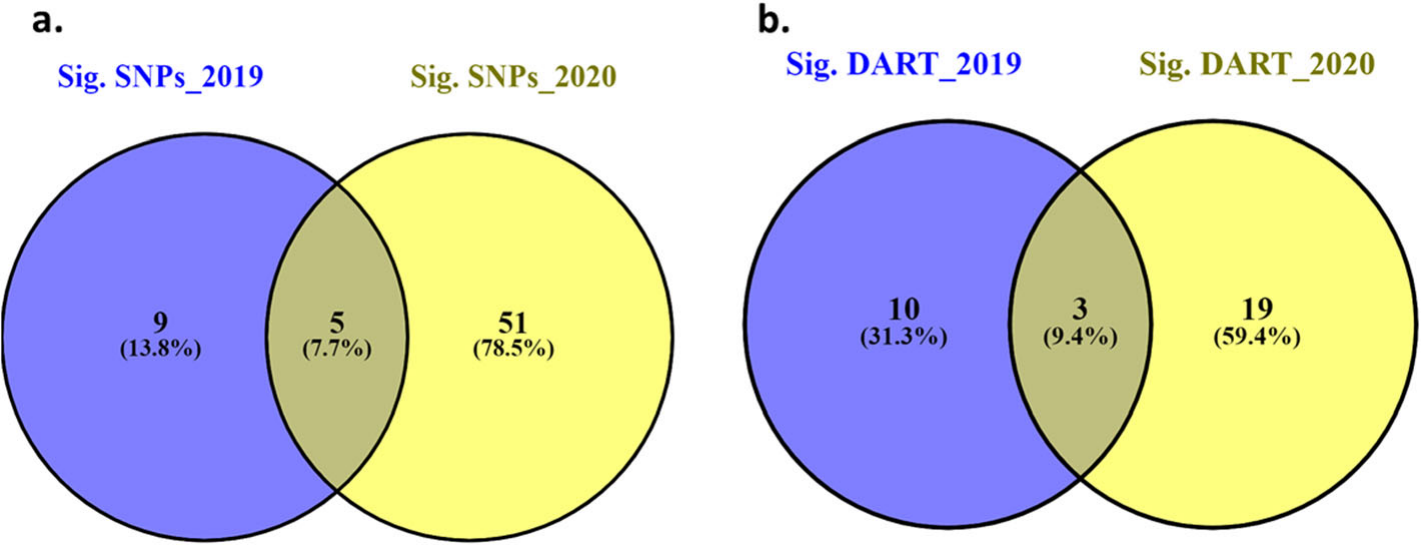
Number of markers significantly associated with the resistance to the Egyptian race of stripe rust at adult growth stage in 2019 and 2020 and the common markers between both years: **a**. significant SNP markers based on the GWAS using GLM+PC model, **b**. significant DArT markers based on single marker analysis (SMA)

**Fig. 5:**
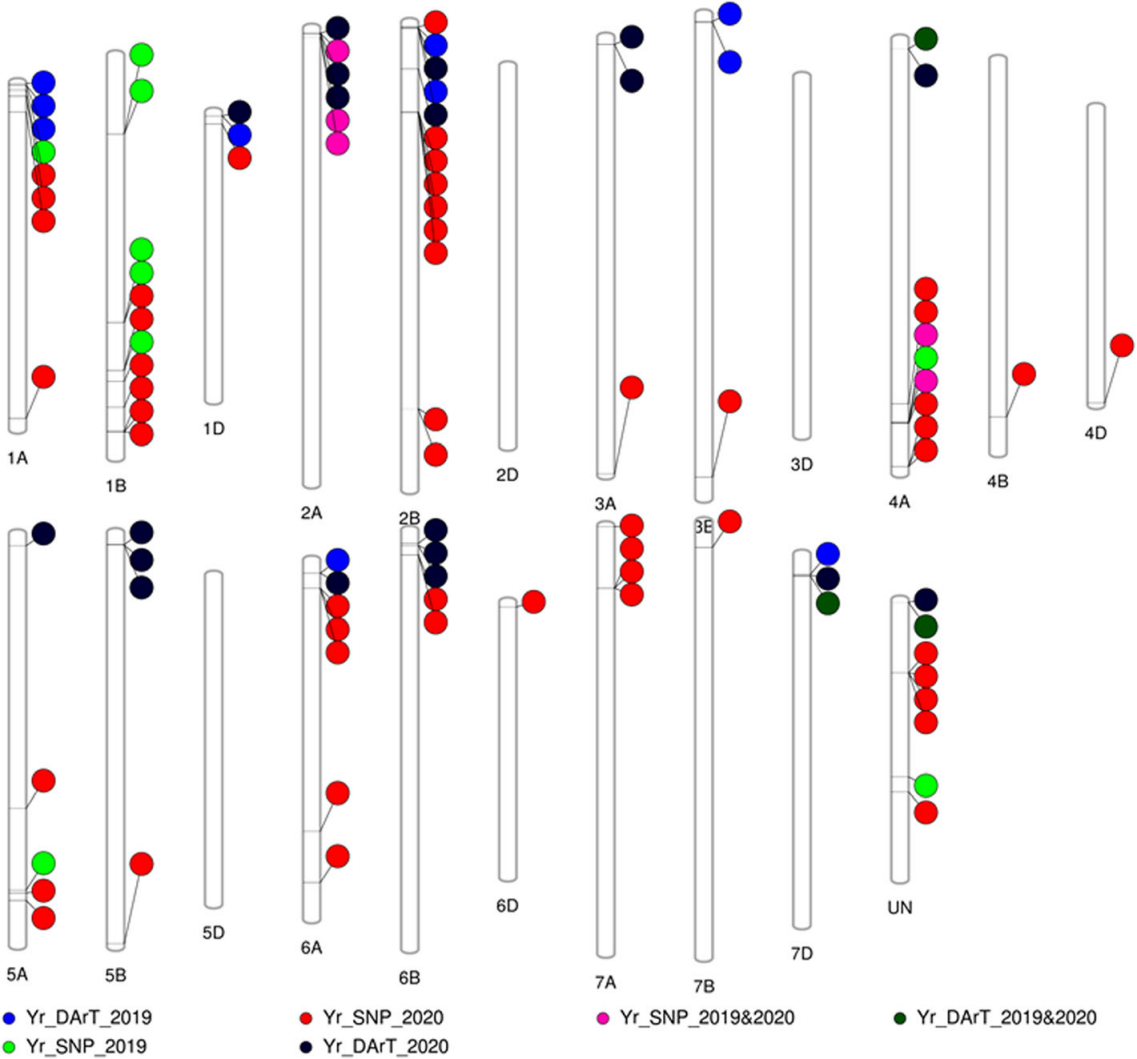
Phenogram represents the chromosomal location of DArT and SNP markers significantly associated with stripe rust resistance at 2019 and 2020 and the common markers between both the two years

Based on the GLM+PC model, a set of 14 and 56 SNPs were identified to be associated with stripe rust resistance in 2019 and 2020, respectively (Figure 4a). The significant SNPs identified in the 2019 experiment were located on chromosomes, 1A, 1B, 2A, 4A, 5A, and unknown chromosomes (Figure 5 and supplementary table 2). While the significant SNPs of the 2020 experiment were located on chromosomes, 1A, 1B, 1D, 2A, 2B, 3A, 3B, 4A, 4B, 4D, 5A, 5B, 6A, 6B, 7A, 7B, and unknown chromosome (Figure 5 and supplementary table 3). Out of the identified SNPs from the 2019 and 2020 trails, five SNPs were common in the two years (Figure 4a). These five SNPs located on chromosome 2A (three SNPs) and chromosome 4A (two SNPs) (Table 3). The phenotypic variation explained by each significant SNPs (R^2^) ranging from 11.03% for SNP marker S4A_658402828 to 23.24% for SNP marker S2A_9121999 in 2019. In 2020, the R^2^ ranged from 14.26% for the SNP marker S2A_16881495 to 30.75% for the SNP marker S2A_9121999. The SNP marker S2A_9121999 had the highest R^2^ in both years with a value of 23.24 and 30.75% in 2019 and 2020, respectively. The allele A of the SNP S4A_658402828 had the highest allele effect which decreases stripe rust symptoms with 55.96% and 65.02 % in 2019 and 2020, respectively. While the allele T in the SNP marker S2A_9121999 had the lowest allele effect which increasing stripe rust resistance with 37.43% and 32.78% in 2019 and 2020, respectively (Table **3**).

**Table 3:** Association analysis of stripe rust resistance using the general linear model + population structure (GLM + PC)

SNP ID	Chro.	-log10 (*P*- value)		Target allele^(1)^	Allele effect ^(2)^		R^2 (3)^ (%)	
		2019	2020		2019	2020	2019	2020
S2A_16067928	2A	6.19	3.18	**G**:C	-46.36	-53.5	11.17	29.89
S2A_16881495	2A	4.19	3.29	**C**:T	-55.17	-42.30	14.19	14.26
S2A_9121999	2A	3.23	3.07	**T**:G	-37.43	-32.78	23.24	30.75
S4A_657492521	4A	3.25	4.78	**G**:A	-40.51	-41.6	11.45	21.96
S4A_658402828	4A	3.29	4.34	**A**:G	-55.96	-65.02	11.03	19.351

^(1)^ The left allele increased the resistance, ^(2)^ The effect of the left allele associated with increasing the resistance, ^(3)^ Phenotypic variation explained by marker

**Fig. 6:**
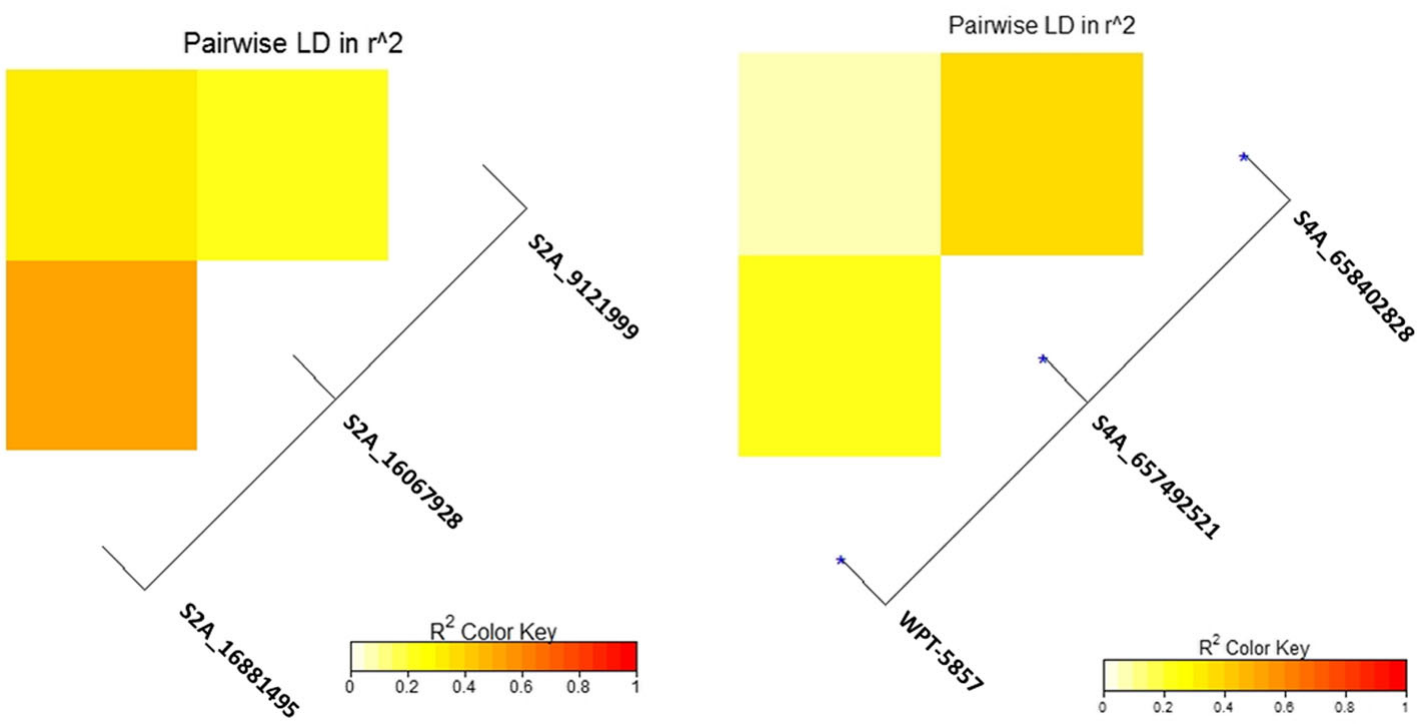
Linkage disequilibrium (LD) analysis in the tested genotypes: **a**. heatmap of LD between the significant SNPs on chromosome 2A and **b**. heatmap of LD between the significant SNPs on chromosome 4A and the significant DArT marker on the same chromosome

The linkage disequilibrium (*r*^*2*^) between each pair of the significant SNPs located on the same chromosome was calculated. For the three significant SNPs on chromosome 2A, no significant LD was found. While an incomplete LD was found for the two significant SNPs on chromosome 4A, with *r*^*2*^ value of 0.51 (Supplementary table 6 and figure 6).

### Genes underlying significant SNPs and their validation

To further understand the genetic association between the significant SNPs and stripe rust resistance in wheat, the annotation of the gene models underlying the significant SNPs was investigated using IWGSC v1.0 GFF3 files. Out of the three significant SNPs on chromosome 2A, two SNPs, S2A_16067928 and S2A_168814950, located within gene models TraesCS2A01G038300 and TraesCS2A01G042100.1, respectively (Table 4). One gene model, TraesCS4A01G380100.1, was found to underly one of the significant SNPs on chromosome 4A.

**Table 4:** Gene models underlying common significant SNPs between 2019 and 2020 and their gene annotations from the International Wheat Genome Sequencing Consortium reference genome assembly v1.0 of the variety Chinese spring

SNP ID	Gene model	Gene annotation	Probable function
S2A_16067928	TraesCS2A01G038300	Beta-glucosidase	Plant defense
S2A_16881495	TraesCS2A01G042100.1	Phosphoglycerate mutase-like protein	Plant adaptation
S2A_9121999	----		
S4A_657492521	TraesCS4A01G380100.1	Coiled-coil domain-containing protein 97	Disease resistance
S4A_658402828	--		

To validate the association between the identified gene models, the functional annotation of these gene models was investigated. TraesCS2A01G038300 gene model is producing Beta- glucosidase, an enzyme which is important in many plant species to improve the plant defense against bacterial, fungus, and insects [38, 39]. Phosphoglycerate mutase protein, produced by TraesCS2A01G042100.1 gene (S2A_16881495), was found to improve the plant adaptation to stresses; mainly abiotic stresses like drought [40]. The coiled-coil domain protein produced by TraesCS4A01G380100.1 gene model was found to have an effective contribution to fungal disease resistance such as powdery mildew in wheat [41, 42].

**Fig. 7:**
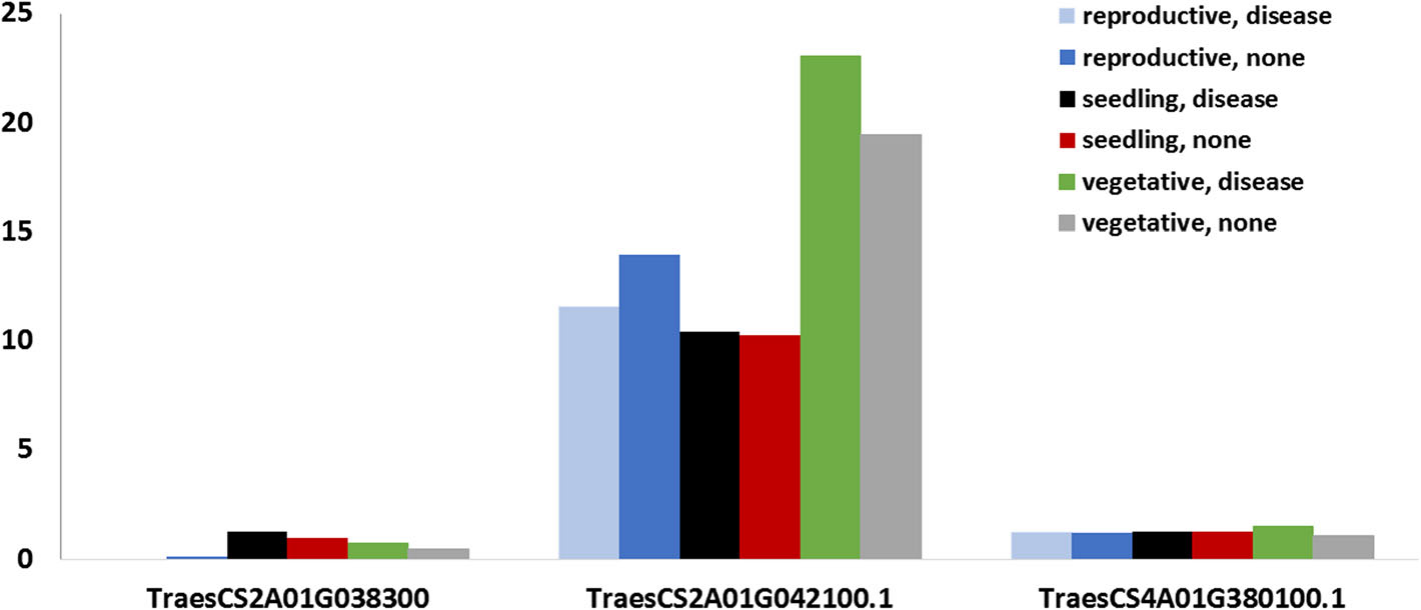
The expression of the three gene models harboring significant SNPs in transcripts per million (TPM). Blue, red and grey column represent the expression under control conditions at reproductive, seedling and vegetative growth stages, respectively. While, light blue, black and green column represent the expression under disease conditions at the same growth stages

Additionally, to robust the association between the significant markers and stripe rust resistance, the expression of the three identified gene models under control and disease conditions at the different plant growing stages: the seedling, vegetative and reproductive stage was compared (Figure 7). All three genes have higher expression under disease conditions compared with the controlled conditions at the vegetative growth stage. Also, the two gene models located on chromosome 2A have a higher expression under the disease conditions compared to the control conditions at the seedling growth stage.

### Single marker analysis (SMA) of stripe rust resistance using DArT marker

SMA identified 13 and 22 significant DArT markers associated with stripe rust resistance in 2019 and 2020 respectively (*p*-value < 0.05) (Figure 4.b and supplementary tables 4 and 5). Out of these significant markers, only three markers were significantly associated with the resistance in both years, 2019 and 2020 (Table 5). The common significant DArT markers are located on chromosomes 1D, 4A, and 7D. The allele effect indicating that two of the three markers, WPT-665480, and WPT-0493, were found to be associated with decrease stripe rust resistance with a percentage of 21 and 27.7 in 2019 and 19.5 and 17.3% in 2020 for each marker, respectively. While the third marker, WPT-5857, was decreasing the symptoms with a percentage of 22.6% and 19.94% in 2019 and 2020, respectively. The phenotypic variation (R^2^) explained by each marker ranged from 5% for marker WPT-665480 to 9% for marker WPT-5857 in 2019 and from 7% for marker WPT-0493 to 16% for marker WPT-5857 in 2020. The LD between the three significant DArT markers and the significant SNPs located on the same chromosome was investigated and no significant LD was found (Figure 6b).

**Table 5:** Single marker analysis (SMA) of the 424 DArT markers for stripe rust resistance in the tested 103 spring wheat genotypes

DArT marker	Chromosome	*-*log10 *(p*-value)		Allele effect		R^2^	
		2019	2020	2019	2020	2019	2020
WPT-665480	1D	1.95160738	3.204069	21	19.5	5%	10%
WPT-5857	4A	1.43955732	1.565027	-22.6	-19.94	9	16%
WPT-0493	7D	1.77392757	1.428376	27.7	17.3	8	7%

### Selection of superior genotypes to stripe rust resistance in the tested materials

**Fig. 8:**
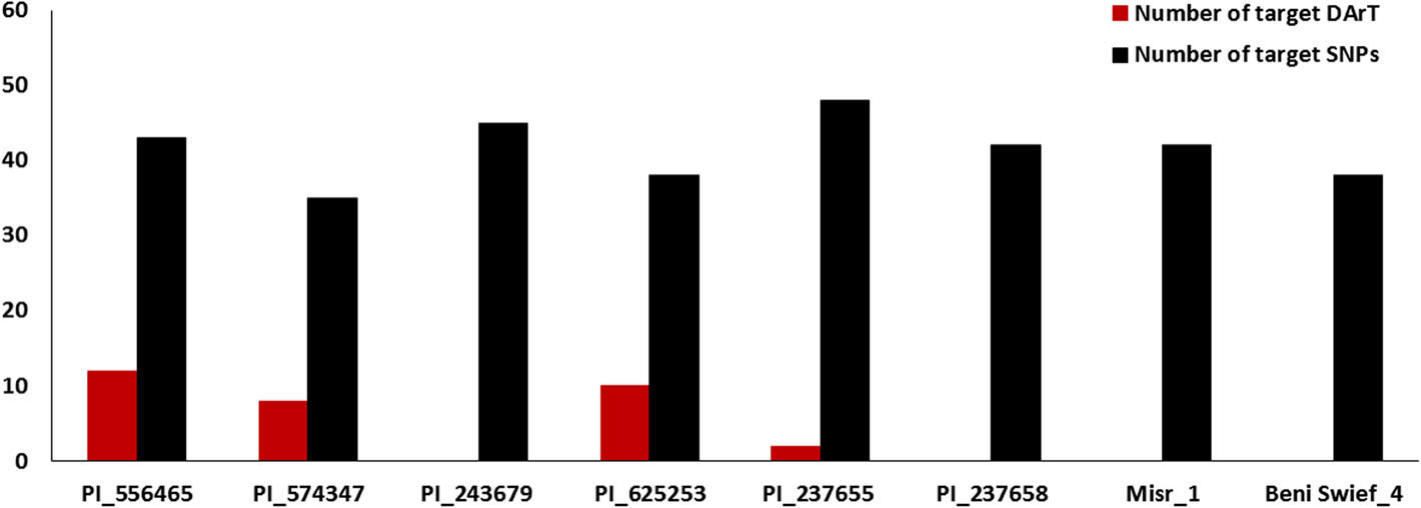
Histogram represents the number of targeted alleles of DArT markers (red columns) and SNP markers (black columns) which were found to be associated with stripe rust resistance in the eight resistant genotypes

To genetically confirm the superior resistance of the promising genotypes presented in Table 2, the number of targeted alleles of all the significant SNPs and DArT markers, either in 2019, 2020, or both years, was investigated in each of the selected genotypes (Figure 8). The Canadian genotype PI_556465 contained a higher number of significant DArT markers (twelve markers). However, four genotypes from the selected eight genotypes did not have available DArT marker genotypic data. As a result, we could not depend on the DArT marker genotypic data to select the best genotypes. For the SNP markers data, the highest number of targeted alleles was found in the genotype PI_237655 from Kenya (48 alleles) followed by the Iranian genotype PI_243679 (45 alleles). The lowest number of targeted alleles (35 alleles) was found in the Saudi Arabian genotype PI_574347. The two resistant Egyptian genotypes contained an intermediate number of targeted alleles with 42 and 38 alleles for Misr_1 and Beni Sweif_4, respectively.

To further understand the possibility of improving stripe rust resistance in the Egyptian genotypes using the currently selected genotypes, the number of different alleles of the targeted SNPs between each pair of the eight genotypes was investigated (Table 6). The number of different alleles ranged from one allele between the Saudi Arabian and Iranian genotypes (PI_574347 and PI_625253) to 15 alleles between the Iranian genotype PI_243679 and both the Egyptian genotype Misr_1 and the Kenyan genotype PI_237658. Comparing between the two Egyptian genotypes and the remaining selected genotypes, the highest number of different alleles was found between both of the two genotypes and the Iranian genotype PI_243679 with 15 and 12 different alleles for Misr_1 and Beni Sweif_4, respectively (Table 6). Also, the genetic distance between the eight selected genotypes was calculated to confirm the success of the crossing between these genotypes. It ranged from 0.5922 between PI_625253 and PI_243679 to 0.7410 between PI_574347 and Beni Swief_4 (Table 6).

**Table 6:** Number of different resistant alleles in the highest resistant genotypes (ND) and the genetic distance between these genotypes (GD)

		PI_556465	Misr_1	Beni Swief_4	PI_574347	PI_243679	PI_625253	PI_237655	PI_237658
PI_556465	ND		9	3	7	11	6	9	9
	GD		0.5985	0.7301	0.6699	0.67139	0.6744	0.6324	0.6571
Misr_1	ND			9	7	15	8	7	0
	GD			0.7039	0.6747	0.6343	0.6318	0.5948	0.6619
Beni Swief_4	ND				7	12	6	9	9
	GD				0.7410	0.7409	0.7015	0.7390	0.6582
PI_574347	ND					3	1	11	7
	GD					0.6861	0.6469	0.6778	0.6423
PI_243679	ND						7	12	15
	GD						0.5922	0.6048	0.6725
PI_625253	ND							6	8
	GD							0.6207	0.6518
PI_237655	ND								7
	GD								0.6516
PI_237658	ND								
	GD								

## Discussion

### Genetic variation in stripe rust resistance in the tested genotypes

Highly significant differences among the genotypes in their stripe rust resistance were found which concluded the success of our phenotypic assay and the presence of a high genetic variation in our tested genotypes (Table 1). The presence of different percentages of CI confirming the high genetic variation which is very useful to select the resistance genotypes under Egyptian environments. Highly significant differences were found between the years and genotypes x years interaction indicating that the response of the genotypes differed in 2019 than 2020. The high degree of broad-sense heritability (H^2^_B_=0.73) indicating that the phenotypic variation in stripe rust resistance is stable and mainly because of genotypic variation. Hence, selection for high resistance genotypes will be successful in future breeding programs. Similar broad-sense heritability degrees were reported in previous studies [43, 44].

Out of the 103-tested genotypes, only eight genotypes were resistant to stripe rust in both 2019 and 2020 with CI ranging from zero to 4%. These eight genotypes representing four different countries, Egypt, Saudi-Arabia, Iran, and Kenya. The diversity among the highly resistant genotypes is very useful in breeding programs as these genotypes can be used as candidate parents to produce cultivars having more resistance to stripe rust against the Egyptian race. Previous studies reported that Beni Sweif_4 is highly resistant to stripe rust infection at the adult stage [45]. Misr_1 was reported to be very resistant and moderate resistance to stripe rust at seedling and adult stages, respectively with very little reduction in seed yield due to the infection [45]. It was postulated to be carrying some major genes such as *Yr5*, *Yr10*, *Yr15*, or *YrSP*. However, pyramiding more resistance genes in these genotypes is required [46]. In our recent study, both the Egyptian genotypes were moderately resistant to stripe rust with a percentage of CI from 2.67 to 4.00 for Misr_1 and zero to 3.33 for Beni Sweif_4 in 2019 and 2020, respectively (Table 2). The presence of a low percentage of infection in the two resistant genotypes confirms that the resistance in these genotypes is broking down and that increasing the level of resistance is urgently needed. Some of the eight resistant genotypes were completely immune to the Egyptian stripe rust race such as PI_243679, PI_237658, and PI_574347 (Table 2). In addition, in 2012 most of our tested genotypes were evaluated to their resistance against stripe rust at Washington State University as a part of the TCAP project and found that genotypes PI_556465, PI_237655, and PI_237658 are moderately resistant (https://tcap.pw.usda.gov/wheat/display_phenotype.php?trial_code=NSGCStripeRust-Spring_2012_MtVernon). These findings confirm the presence of resistant genes in the tested genotypes and concluded that hybridization between these eight resistance genotypes will be very useful in pyramiding multiple resistance genes. However, more understanding of the genetic content of these genotypes is needed.

### Genome-wide association study for stripe rust resistance

Two different models were used to study the association of stripe rust resistance: GLM+PC and MLM+K. Unlike the QQ-plot of the MLM+K model, the QQ-plot of the GLM+PC model represented an ideal distribution on the reference line for both years. This result reveals the importance of the GLM+PC model to identify the candidate regions controlling the inheritance of stripe rust resistance. Similar results were found by Turuspekov et al., (2016) in their studying of stem rust resistance in barley indicating the importance of testing both the MLM and GLM models in the GWAS [47].

Out of the identified significant SNPs in 2019 and 2020, only five SNPs were common between the two years. These five significant SNPs could be considered as stable QTLs for stripe rust resistance in spring wheat. The presence of significant SNPs controlling the resistance in only one year confirming the importance of evaluating wheat genotypes for stripe rust resistance in more than one year to identify the stable SNPs across years. The five stable SNPs are located on two chromosomes (2A and 4A) with R^2^ greater than 10% indicating that all the five significant SNPs are controlling major QTLs for stripe rust resistance in the studied materials. All QTLs that explain more than 10% of the total variation is considered major QTLs [20, 48]. Previous studies identified QTLs on chromosomes 2A and 4A associated with stripe rust adult plant resistance [28, 29].

The absence of significant LD between each pair of the significant SNPs on chromosomes 2A and 4A indicating that the significant SNPs are not coinherited together and the presence of different QTLs on both chromosomes. Many stripe rust resistance genes were mapped on chromosome 2A such as; *Yr1* [49]*, Yr17* [50]*, Yr32* [51], *YrZM175* [52], *Yr56*, *Yr16*, and *Yr48* [53]. Some of these resistance genes were reported to resist the Egyptian race of stripe rust at the adult stage such as *Yr1*, *Yr17*, and *Yr32* [54]. Some stripe rust resistance genes were mapped on chromosome 4A such as *Yr51* and *Yr60* [55]. However, no available information about the reaction of these two genes against the Egyptian stripe rust race. Besides, many studies mapped novel QTLs controlling adult stage stripe rust resistance to this chromosome which confirming our findings [55–57]. More studies are needed to identify the resistance genes on these two chromosomes.

Validation of the association between the identified significant SNP markers and stripe rust resistance

To further understand the association between the significant SNPs and stripe rust resistance in the tested genotypes, gene models harboring the significant SNPs were investigated. Two and one gene models were detected on chromosome 2A and 4A, respectively. Previous studies identified the functional annotation of these three gene models to be related to disease resistance and stress tolerance which confirming our results [43, 58, 59]. The expression of the identified gene models was compared under controlled and disease conditions. The higher expression under disease conditions of the three identified gene models at the vegetative growth stage confirms the effectiveness of the identified genes in the adult plant resistance of stripe rust. In addition, the higher expression of the two gene models located on chromosome 2A at the seedling stage under disease conditions concluded that the identified SNPs on this chromosome might provide a wide range of stripe rust resistance against the Egyptian race in both seedling and vegetative growth stages.

The functional annotation of these gene models confirming the association between the significant SNPs and stripe rust resistance in wheat. Therefore, these five SNPs can be converted to Kompetitive allele-specific (PCR) markers (KASP) and used for validation in different genetic backgrounds.

### Single marker analysis (SMA) of stripe rust resistance using DArT marker

Thirteen and 22 DArT markers were found to be significantly associated with stripe rust resistance in the tested genotypes. Out of these markers, three markers were stable across the two years. All the stable three markers were found to controlling minor QTLs as they have R^2^ value less than 10%. Based on the allele effect, only WPT-5857 markers were found to increase the resistance while the other two markers were decreasing it. Based on Maccaferri et al., (2015) study, this marker (WPT-5857) was mapped on chromosome 4A near 5 QTLs controlling the resistance against the U.S. stripe rust races and near from *Yr51* gene (https://wheat.pw.usda.gov/cgi-bin/cmap/viewer?mapMenu=1&featureMenu=1&corrMenu=1&displayMenu=1&advancedMenu=1&ref_map_accs=Wheat_Yr_genes_and_QTL_4A&sub=Draw+Selected+Maps&ref_map_set_acc=Wheat,%20Yr%20genes%20and%20QTL%204A&data_source=GrainGenes&highlight=%22wPt-5857%22&label_features=all). This marker explained the highest percentage of the phenotypic variation in both years (R^2^) with the highest allele effect (Table 5). The *Yr51* gene is an adult stripe rust resistance gene that is effective against a wide range of Australian and Indian races [60]. In addition, the DArT marker WPT-5857 was mapped within a QTL controlling stem rust resistance in wheat [61]. Therefore, this marker could be useful for marker-assisted selection to improve wheat stripe rust resistance against the Egyptian race.

In addition, we investigated the LD between this DArT marker and the other significant SNPs located on the same chromosome, and no LD was found. From the SMA and the LD results, we can conclude that chromosome 4A carrying more than one QTL/gene which is controlling stripe rust resistance against the Egyptian races. One of these genes might be *Yr51* (Figure 6.b.). For the other two significant DArT marker WPT-665480 and WPT-0493, no previous studies illustrated the association between them and stripe rust resistance in wheat.

### Selection of superior genotypes to stripe rust resistance in the tested materials

To confirm the superior resistance of the high resistance genotypes represented in Table 2, the genetic markers of these genotypes were investigated. Unfortunately, there are no available DArT markers for all the eight resistance genotypes. However, SNP markers are available for all eight genotypes. Each genotype of the eight superior genotypes was examined for the number of the targeted allele of the significant SNPs that it contains. The two resistant Egyptian genotypes contained an intermediate number of targeted alleles, confirming that stripe rust resistance could be improved in the Egyptian wheat germplasm using the current studied materials by crossing with genotypes containing more targeted alleles.

To detect the best genotypes which could be used in future breeding programs to improve stripe rust resistance in Egyptian wheat germplasm, the number of different alleles between each pair of the superior eight genotypes was investigated. The highest number of different alleles was found between the Iranian genotype PI_243679 and the two Egyptian genotypes Misr_1 and Beni Sweif_4. This Iranian genotype was immune against stripe rust Egyptian race in both 2019 and 2020 experiments which confirming our GWAS results and indicating the possibility of increasing stripe rust resistance in the Egyptian genotype using this genotype. PI_243679 was evaluated in the U.S.A. and showed moderate resistance (MR) to a susceptible reaction to the American stripe rust races confirming that the resistance against the Egyptian and U.S. races is controlled by different genetic systems (https://npgsweb.ars-grin.gov/gringlobal/descriptordetail.aspx?id=65098).

Based on our previous population structure analysis, the selected Iranian genotype PI_243679, and the two Egyptian genotypes were located in three different sub-populations [36]. The genetic distance was 0.634325081 between it and Misr_1 and 0.740934344 between it and Beni Sweif_4. Previous studies concluded that crossing between high genetically distant genotypes produces lines with distinct alleles controlling the trait and high combining ability [62]. The high number of different alleles, high genetic distance as well as the distribution among different sub-populations confirming that PI_243679 is the best parent which could be used in crossing with Egyptian genotypes to improve stripe rust resistance in wheat.

## Conclusion

In conclusion, the high variation in the response of the tested genotypes to the Egyptian race of stripe rust indicating the possibility to select stripe rust-resistant genotypes using the current plant materials. Significant SNPs on chromosome 4A are novel SNP markers to the resistance. The identified significant SNPs could be a reliable source for marker-assisted selection (MAS) and genomic selection for stripe rust resistance after validating them in a different genetic background. The most resistant genotypes for stripe rust were identified based on the coefficient of infection and the number of target alleles. Based on the genetic diversity study conducted on the same set of genotypes, PI_243679 genotype could be considered as the best genotype in the tested materials which could be used as a parent in future breeding programs to improve stripe rust resistance in Egyptian wheat genotypes.

## Methods

### Plant materials

To understand the genetic control of the stripe rust resistance in spring wheat, a set of 103 worldwide genotypes was used. The seeds of these genotypes were obtained from the USDA-ARS, Aberdeen, ID, U.S.A., and collected from fourteen different countries around the world. Out of the tested genotypes, 17 Egyptian genotypes, presenting old and new cultivars, were used. More information about the studied wheat genotypes is available in our previous manuscript [36] and supplementary table 1.

### Stripe rust evaluation

The 103 studied genotypes were evaluated for their resistance to stripe rust under natural infection in two experiments. The experiments were carried out in Sids Agricultural Research Station, for two years (2019 and 2020). The experimental design was a randomized complete block design (RCBD) with three replications. In each replication, each tested genotype was represented in a single one-meter row with 30 cm row spacing. The experiment was surrounded by a spreader area planted with a mixture of highly susceptible varieties; Morocco, Thatcher, and *Triticum spelta*. The spreader plants were inoculated artificially using a mixture of urediniospores of the common Egyptian yellow rust races.

Disease severity (DS) was recorded when the susceptible checks were completely covered with the spores. It was expressed as a percentage coverage of leaves with rust pustules [63]. Infection type (IT) was recorded according to Stakman et al., [64] scale (I, R, MR, MS, and S) as described in Roelfs et al., [65]. The infection type was then converted to numeric scale using the following factors: Immune (0) = 0.0, Highly resistance (R) =0.2, Moderately resistant (MR) = 0.4, Methothetic (X) = 0.6, Moderately susceptible (MS) =0.8, Susceptible (s) = 1.0. After that, DS was multiplied by the converted IT scale (numeric) to get the coefficient of infection (CI). Based on the IT, genotypes were considered resistant if they showed ITs MR, R, or Immune. In addition, the resistant genotypes have a percentage of DS 10% or less. As a result, in our recent study, we considered the genotypes with CI 4% or less as a resistant genotype (10% DS x 0.4 (MR)). Genotypes showed CI more than 4% were considered as susceptible genotypes.

### Statistical analysis of stripe rust resistance

To improve the normality of stripe rust resistance data, the coefficient of infection was transformed using arcsine root square using Excel 2013. Shapiro-Wilk normality test was done to confirm that the transformed data was improved compared with the original data. Analysis of variance (ANOVA) was performed using R software (R Core Team, 2017) using the following model:

$$ {\mathrm{Y}}_{\mathrm{i}\mathrm{jk}}=\mu +{\mathrm{g}}_{\mathrm{i}}+{\mathrm{r}}_{\mathrm{j}}+{\mathrm{y}}_{\mathrm{k}}+{\mathrm{g}\mathrm{y}}_{\mathrm{i}\mathrm{k}}+{\mathrm{e}}_{\mathrm{i}\mathrm{jk}} $$

Where Y_ijk_ is an observation of genotype *i* in replication *j* which was planted in year *k*, μ is the general mean; g_i_ and y_k_ are the main effects of genotypes (fixed effects) and replications (random effects), respectively; e_ijk_ is the error. The broad-sense heritability (H^2^) was calculated as follows:

$$ {H}^2={\sigma}_G^2/\left({\sigma}_G^2+\frac{\sigma_R^2}{ry}\right) $$

where ${\sigma}_G^2$ and ${\sigma}_R^2$ are the variance of the lines and the residuals, *r* is the number of replicates within the experiment, and *y* is the number of years.

### DNA extraction, genotyping-by-sequencing (GBS), and DArT markers

DNA was extracted from all the 103 tested genotypes for the GBS purpose using BioSprint DNA Plant kits (Qiagen, Hombrechtikon, Switzerland) from 2 to 3 leaves of two-week-old seedlings. Genotyping-by-sequencing (GBS) was done using Poland protocol [14] as described in [20]. The SNP calling was done using TASSEL 5.0 software [66]. Chinese Spring genome from the International Wheat Genome Sequencing Consortium (IWGSC) Reference Sequence v1.0 was used as the reference genome in SNP calling as it was extensively described in [20]. The generated SNP markers were filtered based on the following criteria; minor allele frequency (MAF > 0.05), maximum missing sites per SNP < 20% and maximum missing sites per genotype < 20% [20, 21, 37]. The heterozygous loci were marked as missing to avoid overestimation of allele effects [20, 21] and the SNPs were re-filtered again using the same criteria.

In addition to these SNP-GBS data, 69 genotypes out of the 103-studied genotypes had available marker data using 424 DArT markers. The data is available on the USDA website https://npgsweb.ars-grin.gov/gringlobal/search”. Mapping of these DArT markers was a part of the QTL-mapping study where stripe rust evaluation was done in the U.S.A. fields under natural infection [25]. Data on these DArT markers are available on the U.S. National Plant Genome system (https://npgsweb.ars-grin.gov/gringlobal/search.aspx). Most of these DArT markers were mapped near or within QTLs associated with stripe rust resistance in wheat.

Genome-wide association study (GWAS), single marker analysis (SMA), and Linkage disequilibrium (LD)

The 103-tested genotypes were examined previously for their presence of population structure and three sub-population were found. More details will be found in Mourad et.al 2020 study [36]. The genetic distance was calculated among the genotypes using a simple matching coefficient by ‘ade4’, R package [67]. The analysis was performed using the SNP markers with the aid of R software [68].

Due to the presence of population structure, GWAS was done using two different models; Mixed linear model + Kinship (MLM+K), and Generalized linear model + principle coordinate (GLM+PC) to identify SNP markers significantly associated with the resistance. The association was done using TASSEL 0.5 software [66]. To identify the significant SNP markers, -log10 of the marker p-value was calculated, and the significant SNPs had a value greater than 3.00. For each significant SNP, the allele which has a negative effect is the targeted allele as it decreases the disease symptoms. On the other hand, the allele with positive values decreases the resistance. Also, phenotypic variation explained by marker R^2^ was calculated for the significant SNPs using TASSEL 5.0 [66]. QQ-plots were developed using ‘qqman’ R package [69]. Significant SNPs were visualized using the Phenogram website “http://visualization.ritchielab.org/phenograms/document”.

Converted CI data as well as the available 429 DArT markers of the 69 genotypes were used to perform single marker analysis (SMA) in order to identify the DArT markers significantly associated with the resistance (*p*-value <0.05 (-log10 >1.3)). Single marker analysis was performed using PowerMarker software V 3.25 [70] using the following model:

$$ \mathrm{Y}=\mu +\mathrm{f}\ \left(\mathrm{marker}\right)+\mathrm{error},\mathrm{where} $$

Y is equal to the trait value, μ is equal to the population mean, and f (marker) is a function of the significant markers. The phenotypic variation explained by each significant DArT marker was estimated using TASSEL 5.0 software [66].

For the SNP and DArT markers located on the same chromosome and significant in both years, linkage disequilibrium (LD) was calculated using TASSEL 5.0 and visualized as a heatmap using ‘LDheatmap’ R package [71].

### Gene models underlying significant SNPs and their validation

To further confirm the GWAS results, we investigate if any of the significant SNPs were in gene models identified in the reference genome assembly published by International Wheat Genome Consortium (IWGSC V.2). The functional annotation of the identified gene models was retrieved from the genome annotation provided by IWGSC and examined for their association with stripe rust resistance. Furthermore, the expression of the identified gene models was compared under the normal conditions and disease conditions at seedling, vegetative, and reproductive growth stages using Wheat Expression Browser “http://www.wheat-expression.com/”.

## Supplementary Information

**Additional file 1: Supplementary Figure 1.** comparison between the untransformed (a. and b.) and transformed data (c. and d.) of stripe rust resistance for the 2019 growing season (.pdf)

**Additional file 2: Supplementary Figure 2.** comparison between the untransformed (a. and b.) and transformed data (c. and d.) of stripe rust resistance for the 2020 growing season. (.pdf)

**Additional file 3: Supplementary Table 1.** list of the genotypes used in this study, country which they are belong to, USDA_ARS Iss and their sub-population. (.exe). **Supplementary Table 2.** Genome-wide association study of stripe rust resistance using the general linear model + population structure (GLM + PC) for the 2019 experiment (.exe). **Supplementary Table 3.** Genome-wide association study of stripe rust resistance using the general linear model + population structure (GLM + PC) for the 2020 experiment (.exe). **Supplementary Table 4.** significant DArT markers associated with stripe rust resistance in 2019 using single marker analysis (SMA) (.exe). **Supplementary Table 5.** significant DArT markers associated with stripe rust resistance in 2020 using single marker analysis (SMA). (.exe). **Supplementary Table 6.** Linkage disequilibrium between the significant SNPs and DArT markers on chromosomes 2A and 4A. (.exe)

**Additional file 4: Sup Figure 3.** Manhattan plot for stripe rust resistance at 2019

**Additional file 5: Sup Figure 4.** Manhattan plot for stripe rust resistance at 2020

## Data Availability

The Sequence dataset used and/or analysed during the current study are available from the corresponding author on reasonable request.

## Abbreviations

GBS: Genotyping-by-sequencing
DArT: Diversity Array Technology
SNP: Single nucleotide polymorphism
GWAS: Genome-wide association study
LD: Linkage disequilibrium
DS: Disease severity
IT: Infection type
CI: Coefficient of infection
SMA: Single marker analysis
IWGSC: International Wheat Genome Consortium
MAF: Minor allele frequency
