# Supplementary material for: Genomic regions associated with stripe rust resistance against the Egyptian race revealed by genome-wide association study
Source: BMC Plant Biol. 2021 Jan 14;21:42. doi: 10.1186/s12870-020-02813-6 (PMC7809828; doi:10.1186/s12870-020-02813-6)
Supplement: Supplementary file 2 — Additional file 2: Supplementary Figure 2. comparison between the untransformed (a. and b.) and transformed data (c. and d.) of stripe rust resistance for the 2020 growing season. (.pdf) [file 12870_2020_2813_MOESM2_ESM.pdf]

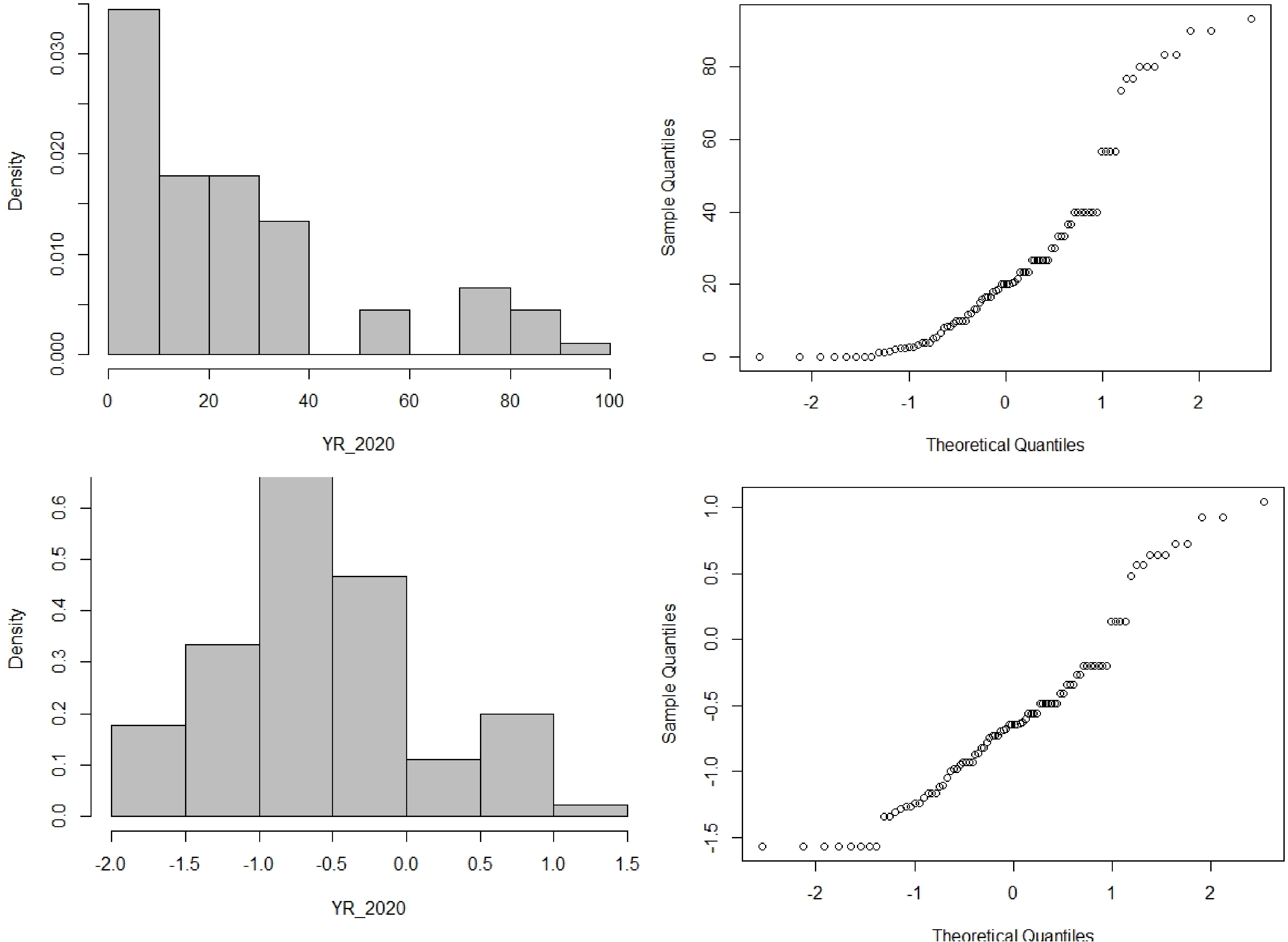

Sup. Figure 2. comparison between the untransformed (a. and b.) and transformed data (c. and d.) of stripe rust resistance for the 2020 growing season
