# Supplementary figures and images for: Genomic regions associated with stripe rust resistance against the Egyptian race revealed by genome-wide association study
Source: BMC Plant Biol. 2021 Jan 14;21:42. doi: 10.1186/s12870-020-02813-6 (PMC7809828; doi:10.1186/s12870-020-02813-6)

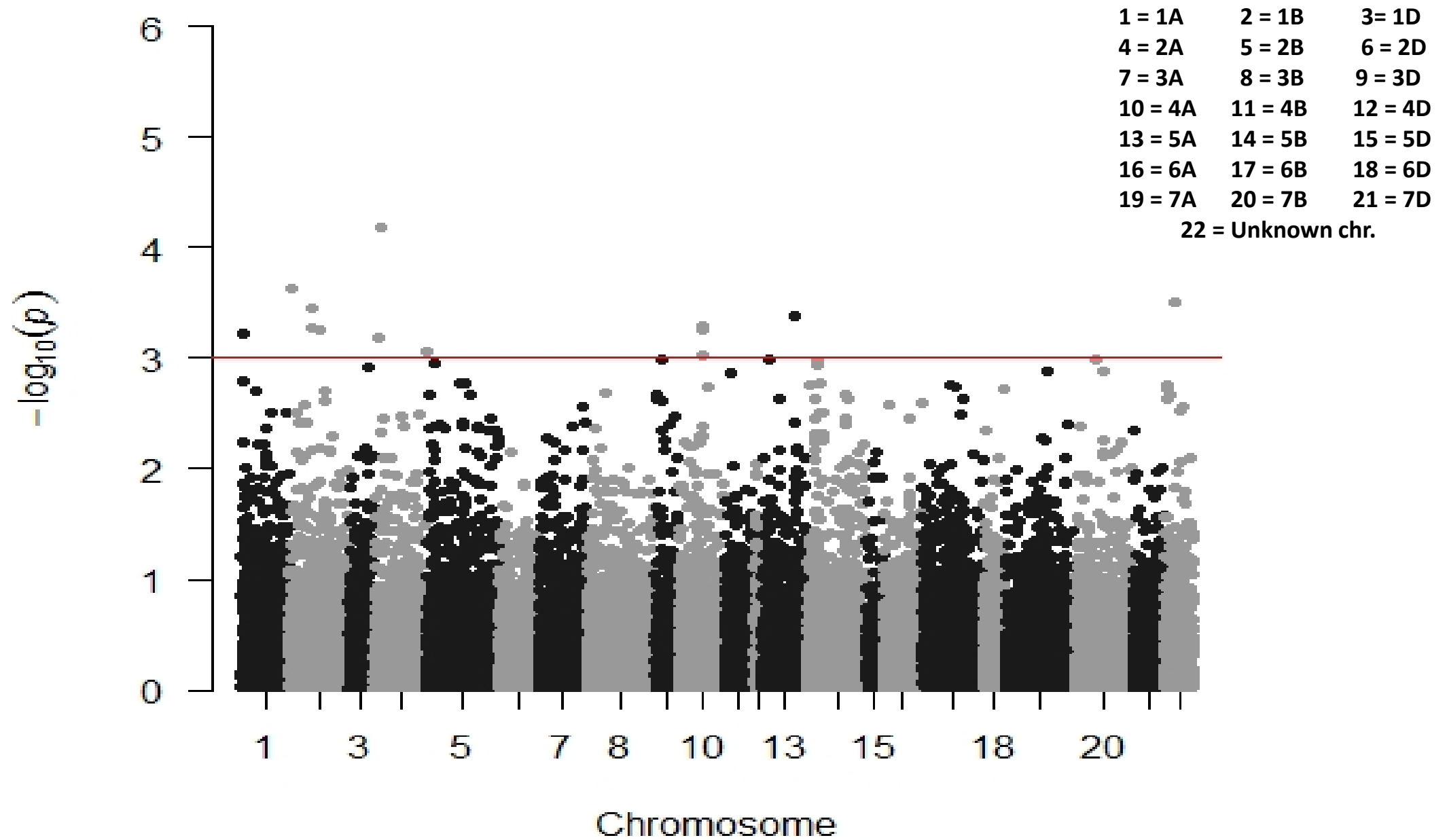

**Sup. Figure 3. Manhattan plot for stripe rust resistance at 2019**

Supplement: Supplementary file 4 — Additional file 4: Sup Figure 3. Manhattan plot for stripe rust resistance at 2019 [file 12870_2020_2813_MOESM4_ESM.pdf]

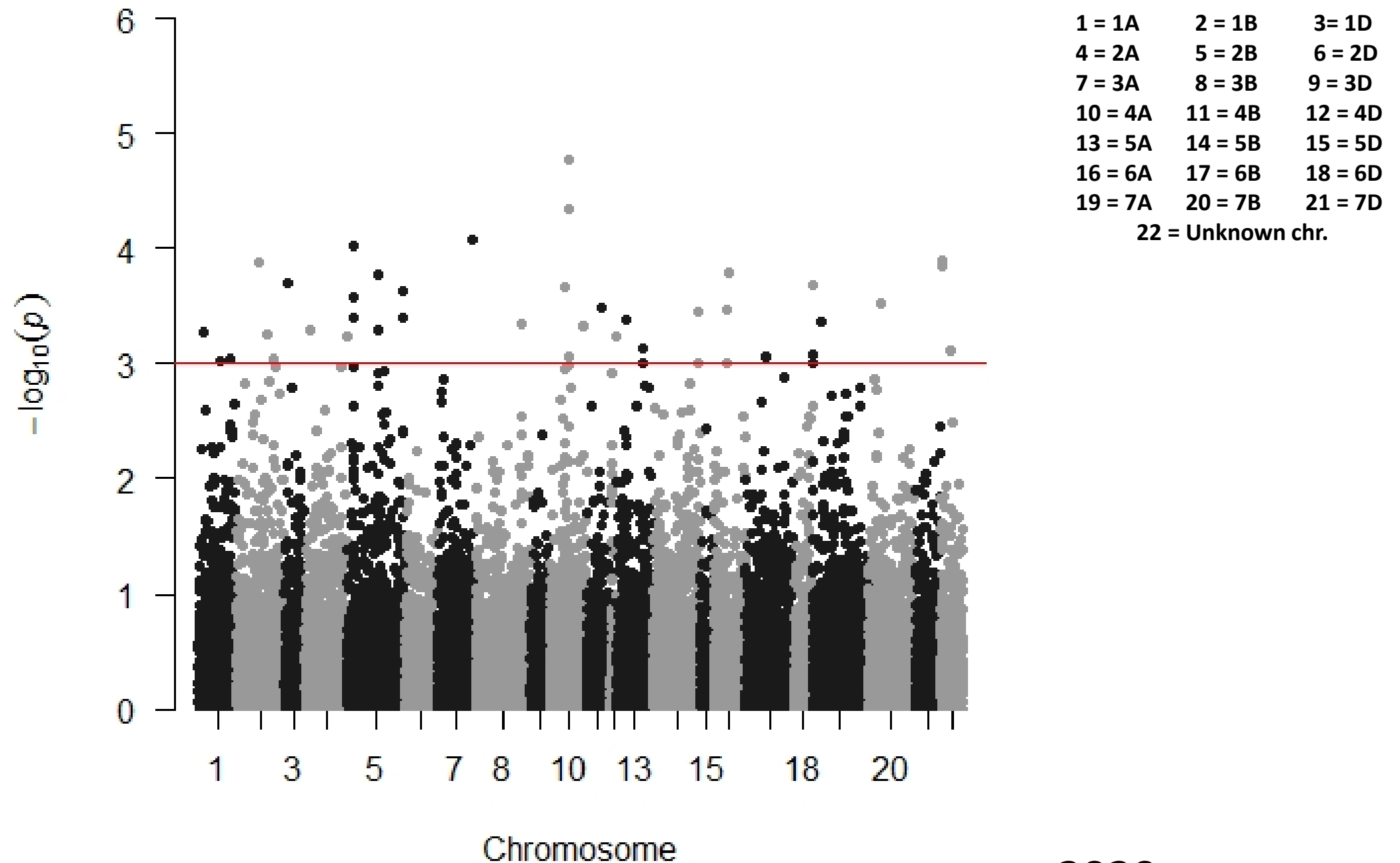

**Sup. Figure 4. Manhattan plot for stripe rust resistance at 2020**

Supplement: Supplementary file 5 — Additional file 5: Sup Figure 4. Manhattan plot for stripe rust resistance at 2020 [file 12870_2020_2813_MOESM5_ESM.pdf]
